# A review on lung disease recognition by acoustic signal analysis with deep learning networks

**DOI:** 10.1186/s40537-023-00762-z

**Published:** 2023-06-12

**Authors:** Alyaa Hamel Sfayyih, Nasri Sulaiman, Ahmad H. Sabry

**Affiliations:** 1 Department of Electrical and Electronic Engineering, Faculty of Engineering, Universiti Putra Malaysia, 43400 Serdang, Malaysia; 2 Department of Computer Engineering, Al-Nahrain University, Al Jadriyah Bridge, 64074 Baghdad, Iraq

**Keywords:** Deep learning, Audio-based diagnosis, Lung sound, Respiratory system, Signal analysis, CNN

## Abstract

Recently, assistive explanations for difficulties in the health check area have been made viable thanks in considerable portion to technologies like deep learning and machine learning. Using auditory analysis and medical imaging, they also increase the predictive accuracy for prompt and early disease detection. Medical professionals are thankful for such technological support since it helps them manage further patients because of the shortage of skilled human resources. In addition to serious illnesses like lung cancer and respiratory diseases, the plurality of breathing difficulties is gradually rising and endangering society. Because early prediction and immediate treatment are crucial for respiratory disorders, chest X-rays and respiratory sound audio are proving to be quite helpful together. Compared to related review studies on lung disease classification/detection using deep learning algorithms, only two review studies based on signal analysis for lung disease diagnosis have been conducted in 2011 and 2018. This work provides a review of lung disease recognition with acoustic signal analysis with deep learning networks. We anticipate that physicians and researchers working with sound-signal-based machine learning will find this material beneficial.

## Introduction

Diagnostics in contemporary medicine are more frequently based on visual or auditory data. Medical knowledge can be obtained in a variety of ways, but to a specialist, it is typically presented as visuals or sounds. It takes time and skill to properly detect health issues based on this information, yet 45% of member states of the World Health Organization (WHO) report having less than 1 doctor per 1000 people, which is the WHO ratio recommendation, according to WHO figures [44]. Given these dismal numbers, the fact that diagnosing entails studying each patient individually over a non-compressible period, and the fact that medical professionals are already overworked, their working conditions are not ideal, and mistakes can be made. The majority of frequent adventitious lung noises heard above the usual signals are crackles, wheezes, and squawks, and their presence typically suggests a pulmonary condition [23, 133, 142]. The traditional techniques of lung illness diagnosis were detected using an Artificial Intelligent (AI-based) method [27] or a Spirometry examination [75], both of which required photos as input to identify the disorders. Going to a hospital to get first analysis by x-ray or chest scan in the event of some Lung suffering condition, such as an asthma attack or heart attack, is time-consuming, expensive, and sometimes life-threatening. Furthermore, the model training over a large number of x-ray images with high-quality HD is required for autonomous an AI-based system of image-based recognition, which is challenging to get each time. A less and simpler resource-intensive system that is able to aid checkup practitioners in making an initial diagnosis is required instead.

This is why it’s important to find new shortcuts for doctors. Automatic and trustworthy tools can assist in diagnosing more patients or they can assist professionals in making fewer errors as a result of work overload. These new tools could come from computer science. For many years, advances in computer science have been steadily enhancing the capacity to autonomously analyze media data in real timing. Diagnosis service techniques should contain the ability to diagnose acoustic or/and visible data. By suggesting quicker and more precise techniques for diagnosis, computer technologies could help nursing personnel or medical experts [28]. The patient could receive adaptable instruments for medical monitoring from it.

Every respiratory examination includes audio auscultation, during which a medical professional uses a variety of instruments (including a sonogram and a stethoscope) to listen to noises coming from the patient’s body. This demonstrates how crucial sound analysis is for identifying lung diseases. Deep learning and machine learning are two new types of techniques that significantly advance the field of audio-based diagnosis [156]. Although less researched, several works analyze respiratory noises [181]. A 2011 review [62] emphasizes that previous studies can identify signs like wheezes or crackles. As earlier declared, the performance of classification and sound detection has significantly increased with the advent of deep and machine learning [42, 43], and research about lung sound analysis has benefited from this development [65, 110, 150]. Lung sound analysis may be converted into a classification problem [29] with the help of identified markers, which is a problem class that machine learning excels at resolving. This seems like a reasonable strategy, although this kind of analysis tends to concentrate more on the characteristics of the sound recording than on the patient level.

The rapid advancement of technology has resulted in a large rise in the volume of measured data, which often renders conventional analysis impractical due to the time required and the high level of medical competence required. Many researchers have offered different AI strategies to automate the categorization of respiratory sound signals to solve this issue. Incorporating machine learning (ML) techniques like Hidden Markov Models (HMM) and Support Vector Machine (SVM) [142], Long Short-Term Memory (LSTM), Residual Networks (ResNet), and Convolutional Neural Networks (CNNs), networks, and Recursive Neural Networks (RNN) are examples of Deep Learning (DL) networks [75]. Deep learning networks are commonly applied as LSTM, Restricted Boltzmann Machines (RBMs), CNN, and Sparse Auto-encoders [152]. In order to extract the relevant features, CNN employs numerous layers of element collections to interrelate the inputs. CNN is used in image recognition, NLP, and recommender systems. Probability distribution within the data collection is learned using RBM. All of these networks train via back-propagation. Gradient descent is used in back-propagation to reduce errors by changing the weights according to the partial error derivative relating to every weight.

The rest of this work is organized as follows: the next subsections provide an overview of breathing sound signals and a list of contributions of this work. “Motivations and problem definition” Section presents a definition of existing problems in the field of lung sound categorization. “Existing Solutions” Section discusses the existing solutions, while “Elaboration Studies” Section discusses the proposed solutions. “Conclusions” Section is represented by the elaboration, which demonstrates algorithms, methods, system components, datasets, and hybrid analysis. Finally, in Sect. 6, the study’s conclusions are presented.

### An overview of breathing sound

**Fig. 1:**
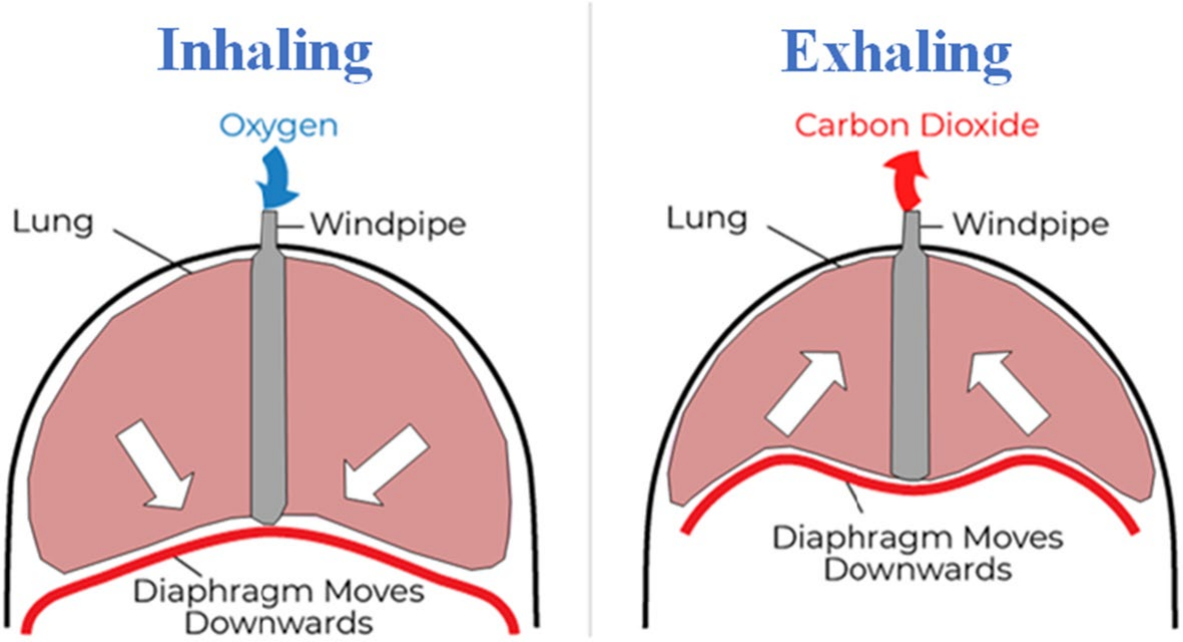
Diaphragm muscles during inhalation and exhalation

Human’s breathing cycle has two distinct phases: inspiration and expiration. Air must be inhaled into the lungs in order to be inspired. The diaphragm drops and its muscles contract during inspiration. As a result, the chest hollow’s volume increases. The hollow in the chest loses air pressure. Outside the body, oxygenated air at high pressure enters the lungs swiftly. The oxygenated air in the lungs travels to the alveoli. The blood vessel network surrounds the slender alveoli walls, which are themselves. Expiration is the process of releasing air from the lungs. The diaphragm rises during expiration as a result of the diaphragm muscles relaxing. As a result, the chest hollow's capacity declines. As a result, carbon dioxide is expelled from the body. Figure 1 provides a demonstration of this procedure.

**Fig. 2:**
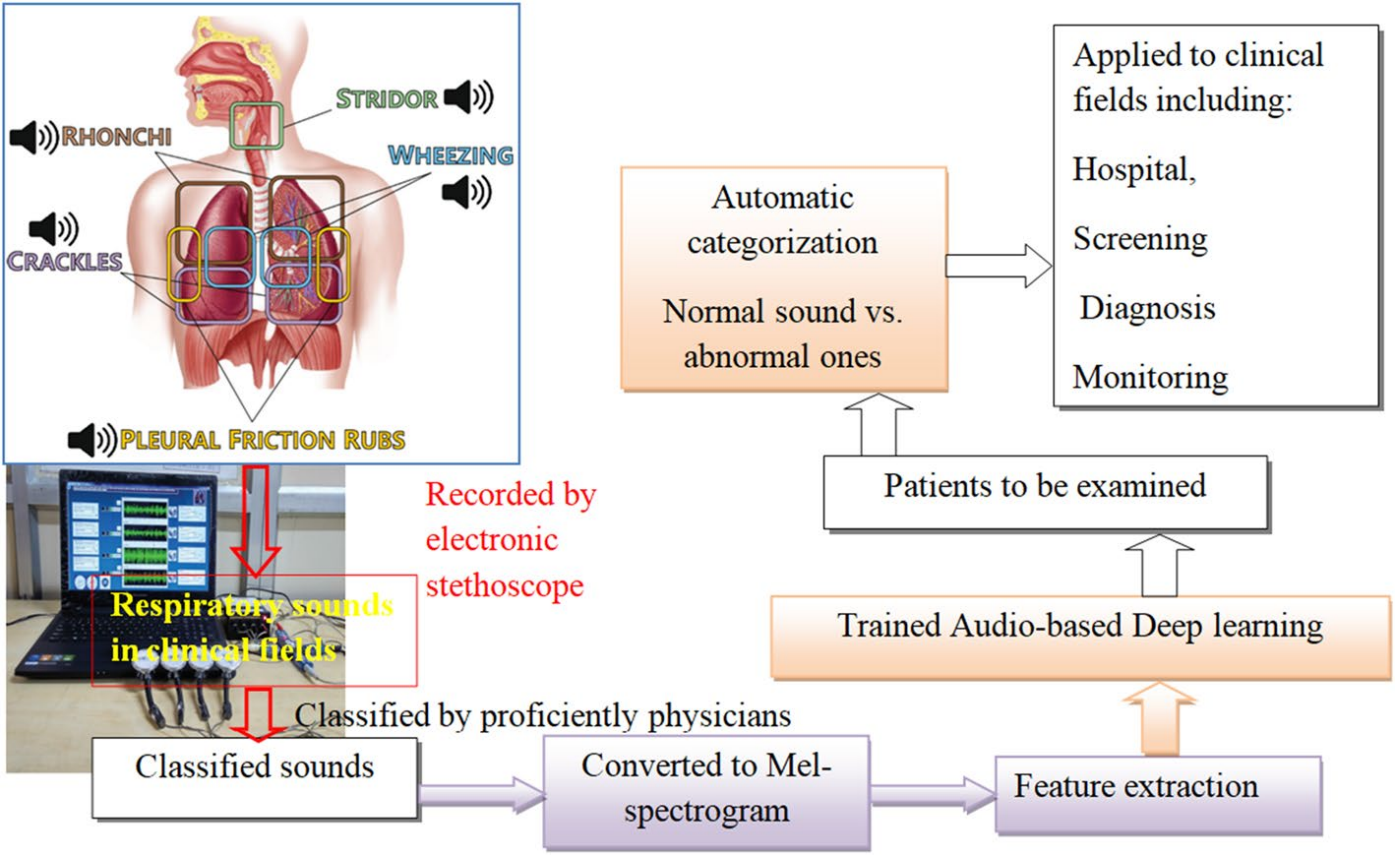
An experimental setup for collecting lung sounds from the back of the chest

An example of an experimental setup to acquire respiratory audio waveforms is illustrated in Fig. 2, where an individual 4-channel audio sensor from four distinct places on the posterior chest of normal and 65 asthmatic individuals was used [72]. The pulmonologist recommended locations that give 66 fewer interfering with heart sounds throughout the lung sound recording procedure.

**Fig. 3:**
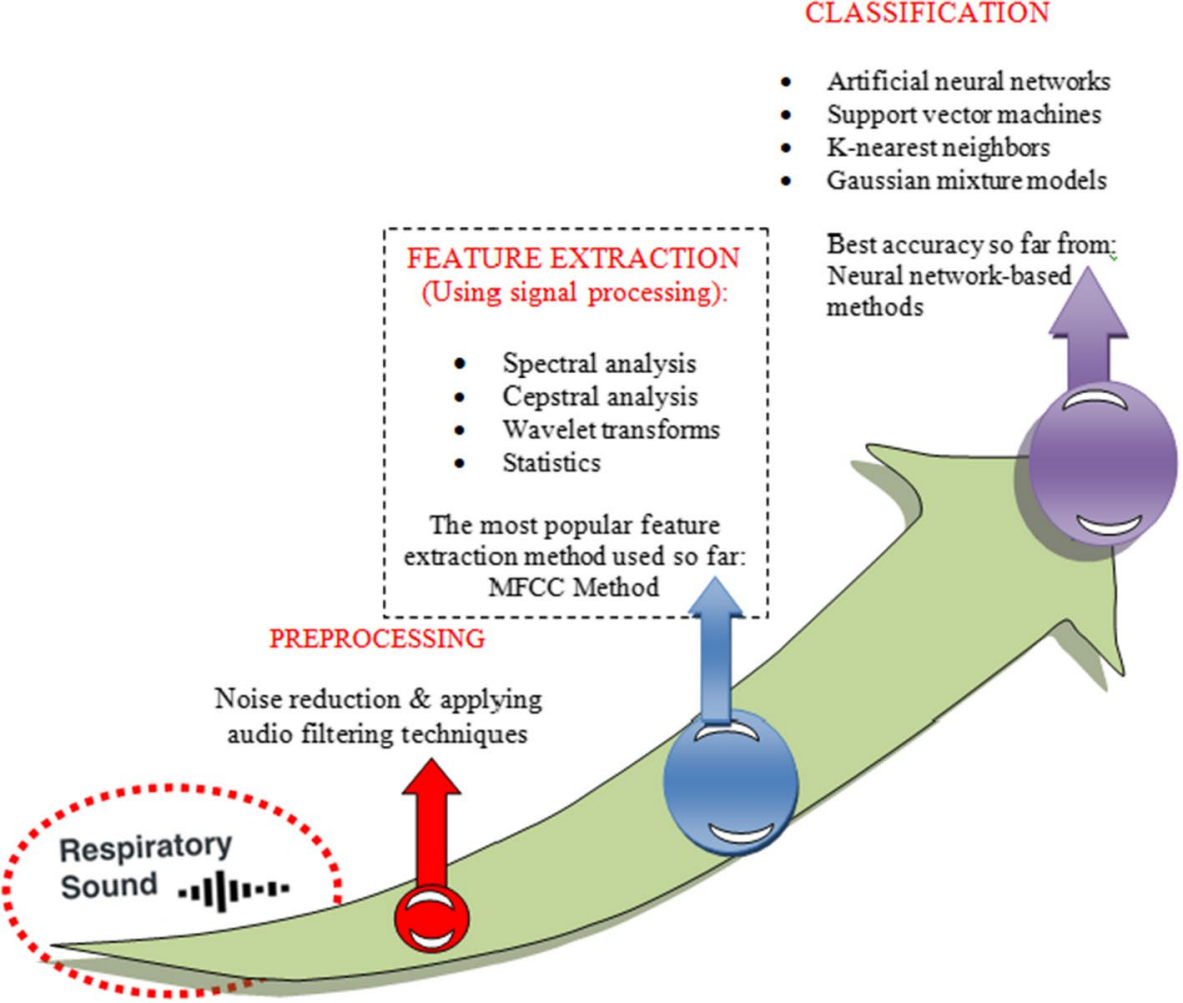
Workflow from preprocessing to classification

Many studies have been conducted feature extraction and selection approaches for computerized lung sound examination and categorization. While conducting feature extraction from a lung sound, entropy-based features, chroma features, wavelet coefficients, Cepstral Coefficients (MFCC), Mel-Frequency and spectrograms are some of the most typically picked features. The deep learning framework employed by the majority of existing work can be generally divided into three stages. The first is respiratory sound preprocessing using audio filtering and noise-lessening methods. The second phase is feature extraction, which is accomplished by the use of signal processing methods such as spectrum analysis [41, 56, 69, 104], Cepstrum analysis [6, 19, 148], wavelet transformations [114, 137, 155], and statistics [113]. The third stage is classification, and the most often used classifiers have been K-nearest Neighbors [34, 63, 116, 127, 158], Support Vector Machines [20, 42, 43, 49, 131, 138], Gaussian Mixture models [105, 107], and ANN [35, 42, 43]. The workflow representation from preprocessing to classification can be shown in Fig. 3.

### Contributions

This review will investigate the algorithms, advances, and diseases applications of sound-based diagnostic techniques of the lung and respiratory systems. Therefore, the characteristics and contributions of this work are as follows:

1. This investigation can help researchers interested in sound-based disease analysis to realize the development trends and characteristics of using such prediction techniques and make sure that they will consciously choose the most suitable algorithms in their research process.
2. The primary trends in prospective medical diagnosis and trends of integrating digital processing are analyzed, revealing that audio-based disease algorithms with deep learning have a shining future.
3. The review searches for the existing problems of lung disease diagnosis with deep learning, such as few samples in the used dataset, poor quality of data, unbalanced data, and poor interpretability, to offer the available appropriate solutions.
4. It presents different forms of comparison tables summarizing recent audio-based deep-learning algorithms in disease classification.
5. Only two review studies based on signal analysis for lung disease diagnosis have been conducted in 2011 and 2018. Therefore, readers will comprehend the methods selection criteria of lung sound handling with large datasets via this paper.

## Motivations and problem definition

Deep learning might be the most significant development in computer science in recent years. Almost all scientific disciplines have been impacted. The world’s leading IT companies and economies are striving to advance deep learning. In a number of sectors, deep learning has already surpassed human performance. This includes diagnosis of obstructive lung disease pattern recognition [38], signal classifiers for cough sound analysis [83], image processing for breast cancer [8], etc. Yann LeCun, Geoffrey Hinton, and Yoshua Bengio, three pioneers of DL, obtained the Award Turing, commonly recognized as the “Nobel Prize” of computers, on 27 of March 2019 [163]. Even if substantial advancements have been made, deep learning still has room for growth. With an additional accurate identification of situations like cancers [15], and the detection of a new medication [11], DL architectures have the prospective to boost human lives. For instance, the authors of the study [9] claimed that DL architectures were able to identify at a similar rank as 21 board-certified dermatologists once learning 2032 illnesses from 129,450 photos. In grading prostate cancer, Google AI could outperform the typical accuracy of USA general pathologists board-certified by 70% to 61% [71].

Only two review studies based on signal analysis for lung disease diagnosis have been conducted on 2011 and 2018. The various deep learning network architectural types, deep learning algorithms for sound-based lung disease diagnosis, their drawbacks, optimization techniques, and the most recent applications and implementations are all included in this review. This review’s objective is to offer a comprehensive overview of scattered knowledge in a single article while covering the large field of deep learning. By assembling the writings of eminent authors from the depth and breadth of deep learning, it delivers innovative work. Other related review publications (see Table 1) concentrate on particular implementations and topics without covering the entirety of the sound/audio-based lung diagnosis.

**Table 1:** Summary of review articles in Lung diagnosis with deep learning networks

References	Publication date	Lung diseases	Types of datasets
[2, 5, 7, 10, 17, 50, 57, 64, 67, 78, 79, 82, 92, 103, 106, 108, 115, 132, 151, 153, 154, 164, 168, 176]	March 2020–December 2022	COVID-19	Images
[58, 145, 160]	2022	COVID-19	Images, Video, audio
[36, 52, 89, 90, 98–100, 126, 136, 162, 166]	January 2019–June 2022	Lung Cancer	Images
[33]	2022	Respiratory health care	Images
[38]	2018	Lung disease	Audio
[62]	2011		
Presented review	2023	Lung disease	Audio

## Existing solutions

The two main approaches used to diagnose the respiratory system are computer-based procedures and clinical methods. Three types of clinical assessment techniques exist classic general examination techniques, history-based techniques, and histopathological image-based techniques. In contrast, there are four main categories into which computer-based diagnosis techniques can be divided: wavelet, image analysis, image processing, and CNN research. Since this technology automatically identifies crucial components without the need for human intervention, we highlight CNN-based audio processing as an exciting area. In this work, we discuss the existing studies in terms of the following problems:

- 1. Dataset Selection: It is essential to obtain and maintain a noise-free database because the entire model is based on it. Preprocessing of the training data must be done correctly.
- 2. Deep learning algorithms choice: Understanding the purpose of the study is important. The best algorithms can be tested to see which ones deliver outcomes that are most similar to the desired outcome.
- 3. Feature extraction strategies: It is a crucial task in the creation of effective models. When high model accuracy is necessary, as well as optimal feature selection, which helps create redundant data during each cycle of data analysis, it is successful.

### Dataset selection

The quality, confidence, and other features of the dataset are essential to measuring the accuracy of training and evaluation of models and architectures that perform on the classification of lung sounds. Several common respiratory/lung sound datasets are listed in Table 2.

**Table 2:** Common respiratory/lung sound datasets in the literature

Dataset name	Description	Used by	Source
Respiratory Sounds Dataset (RSD) [68]	Regular sound signals in addition to three kinds of adventitious respiratory sound signals: wheezes, crackles, and a combination between wheezes and crackles	[16, 53, 54, 59, 114, 117, 129, 139, 171]	[68]
HF_Lung_V1	Comprises 9765 lung sound audio files (each lasting 15 s), 18,349 exhalation labels, 34,095 inhalation labels, 15,600 irregular adventitious sounds’ classes, and 13,883 regular adventitious sound classes (including, 4740 rhonchus classes, 8458 wheeze classes, and 686 stridor classes)	[66]	[66]
Respiratory-database@TR	Each patient has 12-channel lung sounds. Short-term recordings, multi-channel analysis, 5 COPD (chronic obstructive lung disease) severity levels (COPD4, COPD3, COPD2, COPD1, COPD0) (At least 17 s). This dataset was considered by	[13]	[12]
Own generated database	The lung sounds were captured using an e-stethoscope and an amplifier linked to a laptop. An e-stethoscope with a chest piece that is touched by the patient and a microphone-based recording sound signals with a 44,100 Hz sampling rate that is attached to signal amplifiers are used in this setup. The amplifier kits extend the signal range to about (70–2000 Hz) with respiratory sounds (with frequency controller and control amplifier) when associated with an earphone (to listen to live records) and a PC	[21]	[21]
Own generated database	Data is separated into two types: Sub-interval set, which includes complete patient set, which comprises all patients' measures and is classed as Abnormal or Normal, counting all patients' sub-interval measurements of any duration. It has around 255 h of measured lung sound signals	[46]	[46]
Own generated database	RSs non-stationary data collection with 28 separate patient records. For training and testing, two distinct sets of signals were employed. Except for crackles and wheezes, which were data from six patients each, each class in the training and test sets comprised two recordings from distinct patients. The sampling frequency of the recorded data was 44.1 kHz	[122]	[122]
R.A.L.E. repository	It is a collection of digital recordings of respiratory sounds in health and sickness. These are the breath sounds that physicians, nurses, respiratory therapists, and physical therapists hear using a stethoscope when they auscultate a patient's chest. Try-R.A.L.E. Lung Sounds, which provides a vast collection of sound recordings and case presentations, as well as a quiz for self-assessment	[22]	[159]
R.A.L.E. lung sounds 3.0	It includes five regular breathing recordings, four crackling recordings, and four wheeze recordings. To eliminate DC components, a first-order Butterworth high-pass filter with a cut-off frequency of 7.5 Hz was employed, followed by an eighth-order Butterworth low-pass filter with a cut-off frequency of 2.5 kHz to band restrict the signal	[4]	[123]
Respiratory sound database	It developed by two Portuguese and Greek research teams. It has 920 recordings. The duration of each recording varies. 126 patients were recorded, and each tape is documented. Annotations include the start and finish timings of each respiratory cycle, as well as if the cycle comprises wheeze and/or crackle. Wheezes and crackles are known as adventitious noises, and their presence is utilized by doctors to diagnose respiratory disorders	[21, 84, 114, 127, 129, 135]	[134]

*R.A.L.E.* refers to *(Respiratory Acoustics Laboratory Environment)*

### Deep learning algorithms for lung sound

Deep Learning CNN (DLCNN) is being used to diagnose obstructive lung illnesses, which is a fascinating development. DLCNN algorithms function by identifying patterns in diagnostic test data that are possible utilization to forecast clinical outcomes or identify obstructive phenotypes. The objective of this work is to present the most recent developments and to speculate on DLCNN’s future potential in the diagnosis of obstructive lung disorders. DLCNN has been effectively employed in automated pulmonary function test interpretation for obstructive lung disease differential diagnosis [53, 54], where all sound data were examined to meet segmented into 5 s segments and 4 kHz sampling frequency. The architecture of the deep learning network contains two steps; bidirectional LSTM units and CNNs. Then, a number of processing steps were implemented to assure less noisy and smoother signals. This includes z-score normalization, displacement artifact removal, and wavelet smoothing. The proposed algorithm classified patients according to the different categories of lung diseases with the greatest precision of 98.85% and average accuracy of 99.62%. For obstructive pattern detection in computed tomography and associated acoustic data, deep learning algorithms such as neural networks using convolutions are state-of-the-art [39]. DLCNN has been applied in small-scale research to improve diagnostic procedures such as telemedicine, lung sound analysis, breath analysis, and, forced oscillation tests with promising results.

Deposits in the respiratory system limit airways and induce blood oxygen deficit, resulting in erratic breathing noises. Obtaining these respiratory sounds from test subjects, extracting audio features, and categorizing them will aid in the detection of sputum or other infections. These sickness stages can be accurately classified using deep learning convolution neural network methods. Several studies reviewed DLCNN such as [51], where its goal was to find the best CNN architecture for classifying lung carcinoma based on accuracy and training time calculations. Backpropagation is the best feed-forward neural network (FFNN) method, with an accuracy of 97.5 percent and training time of 12 s, and kernel extreme learning machine (KELM) is the best feedback neural network (FBNN) method, with an accuracy of 97.5 percent and an 18 min 04 s training time.

The majority of studies in the literature used numerous classifiers to see which one produced the greatest accuracy results that are regarded as a main performance metric of study. DLCNN methods such as VGG (VGG-B3, VGG-B1, VGG-V2, VGG-V1, and VGG-D1), Res-Net, LeNet, Inception-Net, and AlexNet, were applied to spectrum data for categorization functions, and the results were analyzed and compared with one another to improve categorization of aberrant respiratory sounds.

**Fig. 4:**
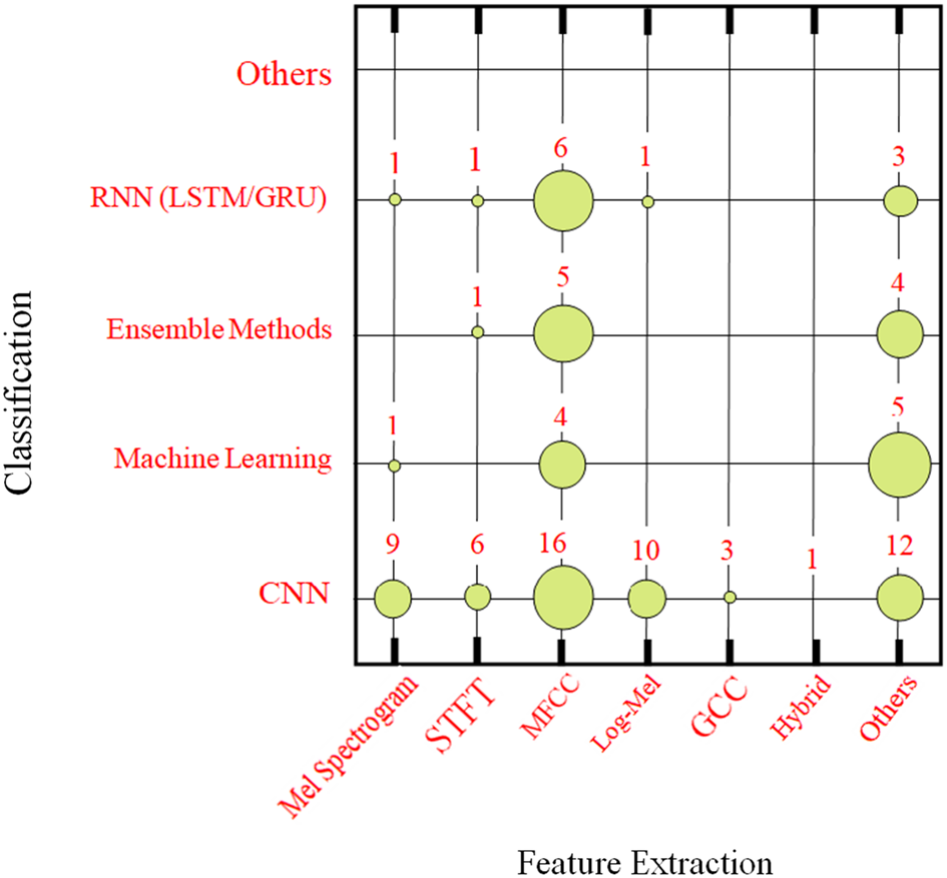
Graphical representation for the number of publications of crossing feature extraction methods with the categorisation in terms of circles of varying diameters

The distribution of publications by classification and feature extraction techniques is shown graphically in Fig. 4, where the majority of studies used CNNs for classification and MFCC for feature extraction. Along with other feature-based techniques that have been sparingly employed with machine learning and ensemble techniques, MFCC was routinely utilized with RNNs, ensemble learning, and machine learning. The main deep learning algorithms for sound-based classification employed in this study are mentioned in Table 3.

**Table 3:** Audio-based categorization using deep learning methods

Application	Classification method	References
Environmental sound classification and acoustic scene classification	ResNet	[45, 70, 97, 112]
	Autoencoder DNN	[98–100, 120]
Snore sound classification, speech emotion recognition, and respiratory sound classification	Gated Recurrent Unit (GRU)	[24, 128, 175]
Various applications	Deep neural network (DNN)	[77, 121, 125, 128, 146]
	CNN	[32, 40, 76, 81, 86, 88, 96, 128, 140, 157, 161, 165, 167, 173, 174, 177, 178, 180]
	Deep CNN (DCNN)	[102, 111, 179]
Sound event detection, speaker detection	Bidirectional gated	[177, 178, 180]
	Recurrent neural	
	Networks (BiGRU)	
Environmental audio classifications	(BEIT) Bidirectional encoder	Wyatt et al. [170]
	Illustration from	
	Transformers	
Sound event recognition	Audio event recognition network (AReN)	Greco et al. [60]
Environmental sound and acoustic scene classification	Sound/event credit network	[45, 47, 112]
Sound event detection	Adaboost	Ykhlef et al. [174]
Sound classification	Using the restricted boltzmann machine, DNN was trained	Ozer et al. [124]

### Feature extraction strategies

**Fig. 5:**
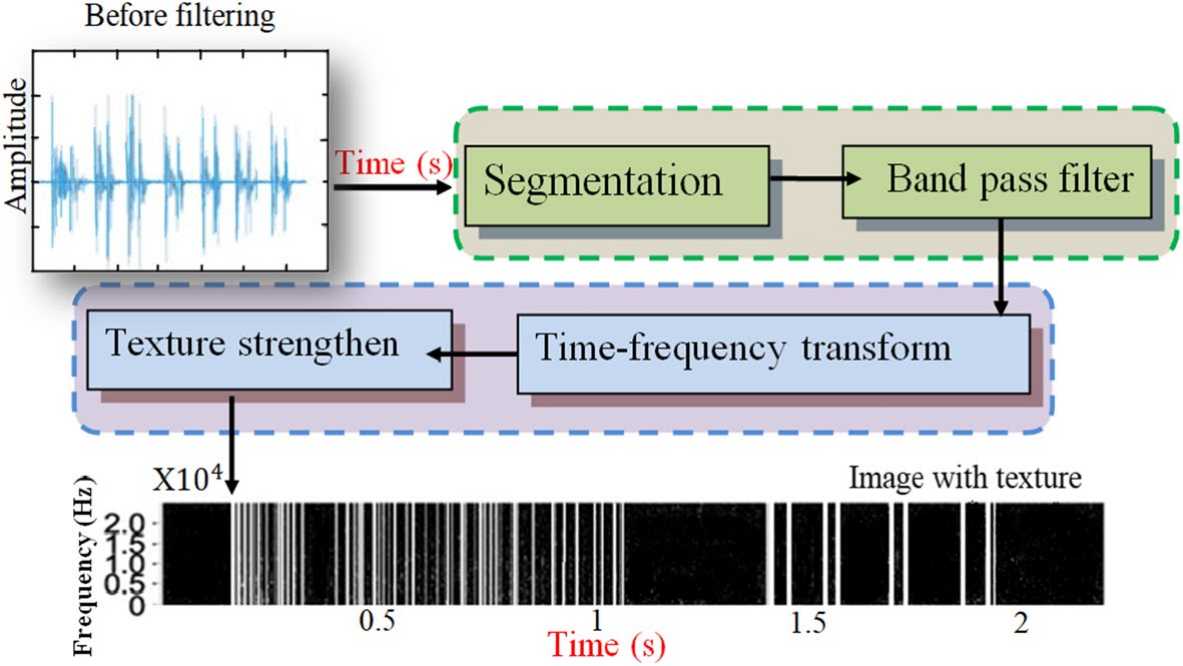
Block diagram of the signal preprocessing stage [119, 147]

Data preprocessing begins with importing the re-sampling, cropping them, and sound files. Because recordings are made by different research teams using different recording equipment, sampling rates vary (4000 Hz, 44100 Hz, and 10000 Hz). All recordings may be re-sampled to a single sampling rate, such as 44100 Hz, and every sound is typically 3–10 s extended by zero-padding shorter segments and cropping larger ones. The respiratory sound data are divided into distinct breaths during preprocessing by detecting the lack of sound between breaths. Lung sounds captured from different participants will have varying loudness levels. As a result, before processing, the signals were adjusted such that they were roughly the same loudness regardless of the subject. Most of the methods from literature normalize a signal before being divided into frequency sub-bands using the discrete wavelet transform (DWT). To depict the allocation of wavelet coefficients, a set of numerical characteristics was collected from the sub-bands. A CNN-based scheme was implemented to classify the lung sound signal into one category: squawk, crackle, wheeze, normal, rhonchus, or stridor. The schematic block diagram of the signal preprocessing stage is described in Fig. 5.

A method for extracting and detecting characters based on lung sounds was described in the paper [149]. The wavelet de-noised approach removes noise from the collected lung sounds before employing wavelet decomposition to recover the wavelet features parameters of the denoised lung sound signals. Because the characteristic vectors of lung sounds have multi-dimensional following wavelet reconstruction and decomposition, a novel technique for converting them into reconstructed signal energy was developed. They also used linear discriminate analysis (LDA) to decrease the length of feature vectors for assessment in order to create a more efficient recognition technique. Finally, they employed a BP neural network to differentiate lung sounds, with 82.5 percent and 92.5 percent recognition accuracy, respectively, using relatively high-dimensional characteristic vectors as input and low-dimensional vectors as output. The study evaluated lung sound data using the Wavelet Packet Transform (WPT) and classification with an artificial neural network [93, 94]. Lung sound waves were separated into statistical parameters and frequency sub-bands using the WPT were derived from the sub-bands to describe the distribution of wavelet coefficients. The classification of lung sounds as normal, wheezing, or crackling is done using an ANN. This classifier was programmed by a microcontroller to construct a portable and automated device for studying and diagnosing respiratory function. In the study [93, 94], a method for distinguishing between two types of lung sounds was provided. The proposed technique was founded on an examination of wavelet packet decomposition (WPD). Data on normal abnormal and normal lung sounds were collected from a variety of patients. Each signal was split into two sections: expiration and inspiration. They used their multi-dimension WPD factors to create compressed and significant energy characteristic vectors, which they then fed into a CNN to recognize lung sound features. Widespread investigational results demonstrate that this characteristic extraction approach has high identification efficiency; nonetheless, it is not yet ready for clinical use. A common procedure to processing the lung sound can be listed as follows:

1. As input, a Lung sound recording folder is used. Lung sounds are a combination of lung sounds and noise (signal interference).
2. As a signal, sounds are able to be played and written.
3. The Lung sounds are then examined by the scheme, saved in the data, and divided into an array of type bytes.
4. The data array is transformed into a double-sized array.
5. Repeatedly decomposing array data according to the chosen degree of disintegration creates two ranges, every half the duration of the data range. The initial array is known as a low-pass filter, while the second span is known as a high-pass filter.
6. Apply the wavelet transform to the coefficients in each array.
7. In the data array, both arrays are reconstructed, with a low-pass filter at the beginning and a high-pass filter at the ending time.
8. The data array is processed via a threshold, creating respiratory sound signal noise and two arrays.
9. Repeat restoration as many times as the stage of restoration set to each array.
10. In the data array, reverse the order of the preceding half high pass filter and half low pass filter, discontinuous high pass filter low pass filter for every array.
11. Re-perform each array's wavelet transform parameters.
12. Data Array is then transformed from a double-sized array to a byte-sized array. The acoustic format and folder names that have been specified are functional to the information.
13. A signal [data] of a breathing sound set is restructured to a breathing sound folder, and a data noise array is restructured to a noise beam.

Wavelet is relation of functions ${\varphi }_{a,b}t$ resulting from a foundation wavelet $\varphi \left(t\right)$, called the “mother wavelet”, by translation and dilation [117] as described in Eq. (1):

1 $$\varphi_{a,n} t = \frac{1}{\sqrt a }\varphi \left( {\frac{t - n}{a}} \right), a > 0,n \in \Re$$

Wavelet examination is essentially scaling and shifting a restricted shape of energy called the “mother wavelet” $\mathrm{\varphi }\left(\mathrm{t}\right)$ of the preferred indication. So, the disconnected wavelet change is able written as follows:

2 $$\varphi_{j,k} \left( t \right) = 2^{\frac{j}{2}} \varphi \left( {2^{j} t - k} \right)$$

The signal-to-noise ratio ($SNR$) is a dimensionless relation of the power of a signal to the associated power noise during recording, this can be expressed by [16]:

3 $$SNR = \frac{{P_{signal} }}{{P_{noise} }} = { }\left( {\frac{{A_{signal} }}{{A_{noise} }}} \right)^{2}$$

where ${A}_{noise}$ denotes root mean square (RMS) of noise amplitude, ${\mathrm{A}}_{\mathrm{signal}}$ represents the root mean square (RMS) of signal amplitude, ${P}_{noise}$ denotes the mean of noise power, and the ${P}_{\mathrm{signal}}$ denotes the mean of signal power.

The studies [93, 147] decomposition after evaluating the distribution characteristics of time–frequency respiratory sounds. The optimum wavelet packet foundation for feature extraction was chosen after the space partitioning of wavelet packets. They can perform quick random multi-scale WPT and get every high-dimension wavelet parameters matrix based on the best basis. The time-domain equal-value relationship between coefficients wavelet and signal energy was then established. The energy was used as an eigenvalue, and vectors of characteristics from a classification artificial neural network (ANN) were used as forms. This drastically reduces the number of ANN input vectors. Extensive experimental results reveal that the proposed feature extraction approach outperforms other approaches in terms of recognition performance. The time-domain equal-value relationship between wavelet coefficients and signal energy was then established. The energy was used as an eigenvalue, and feature vectors from a classification artificial neural network (ANN) were used as forms. The number of ANN input vectors is considerably reduced as a result. Extensive experimental findings show that in terms of recognition performance, the suggested feature extraction technique surpasses alternative approaches.

**Fig. 6:**
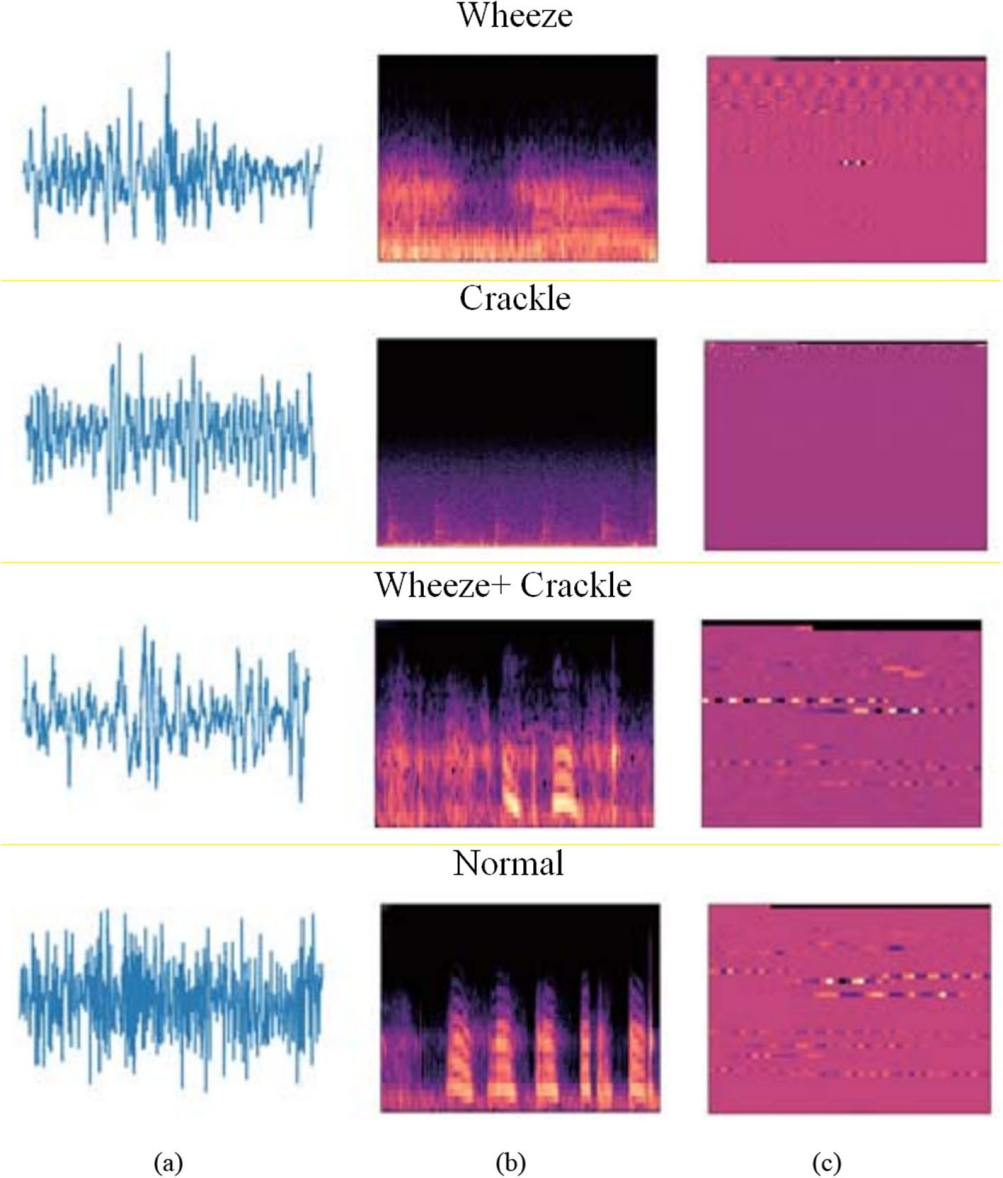
(**a**) Original sound signal. (**b**) STFT spectrogram (**c**) wavelet generated spectrogram of lung sounds. From respiratory sound database [68] Challenge | ICBHI Challenge, n.d.)

To provide a clear insight on features extractions of lung sound, we downloaded free samples of lung sounds from the [68] database [68]* Challenge | ICBHI Challenge*, n.d.) and performed both wavelet analysis and short-time Fourier transform (STFT) as a two different algorithms. The original waveforms are shown in Fig. 6a for Wheeze, Crackle, Wheeze + Crackle, and normal sound. For Wheeze signals, the prevalence of the spectrum power falls within the (100–1000) Hz frequency range, with a particular transient of shorter than 100 ms. Crackle signals have an oscillatory signature with (350–650) Hz frequency range and it lasts more than 20 ms. Figure 6b shows the STFT spectrogram for every respiratory segment. The wheeze and crackle signals are likewise supplied in the same records of the [68] database. Since the combined signals are frequently asymmetric and erratic, it can be challenging to isolate and identify the essential component from the STFT spectrums. In order to increase the accuracy of deep learning model, the study [101] additionally used the wavelet packet analysis. The wavelet generated spectrogram is shown Fig. 6c.

**Fig. 7:**
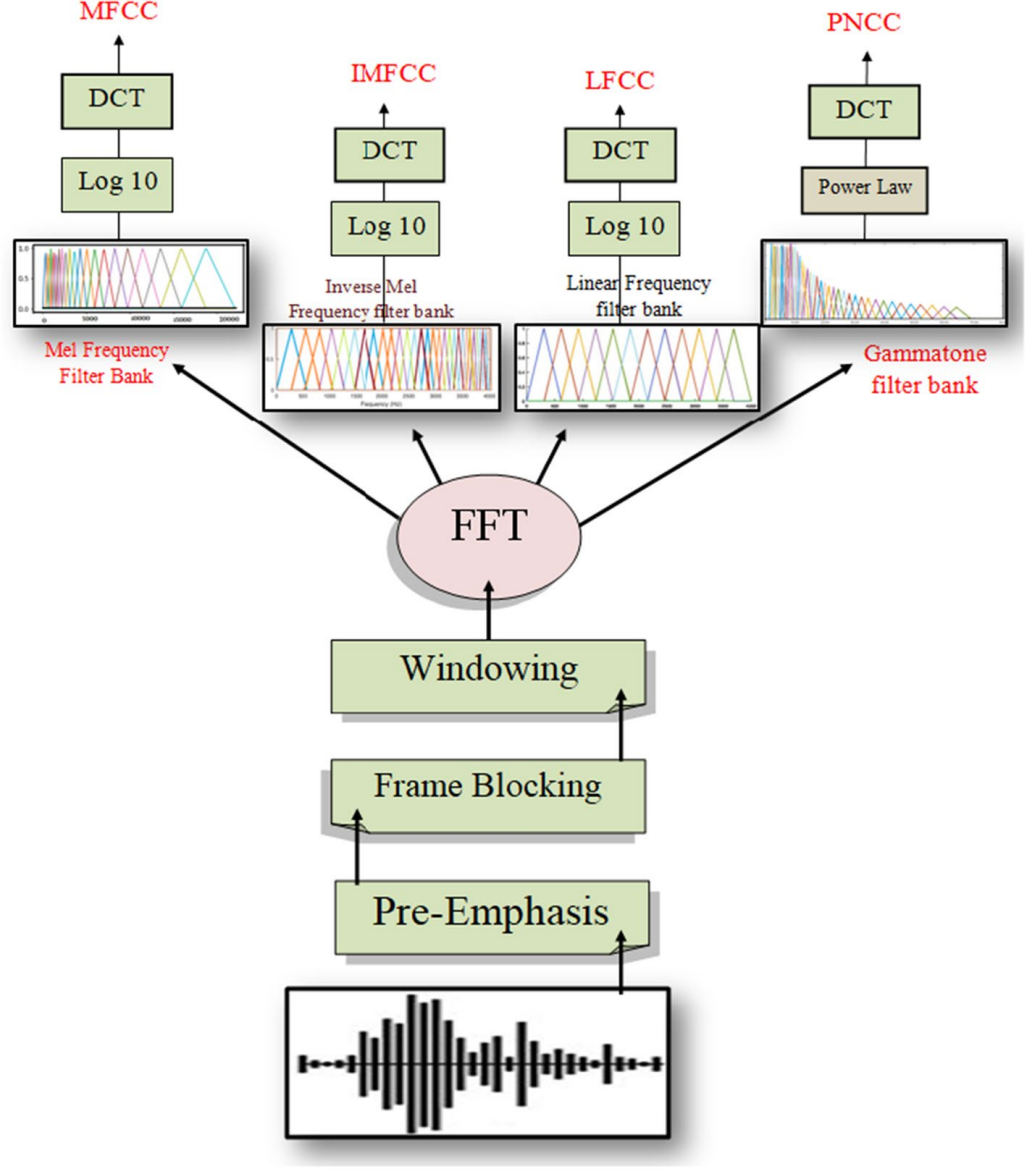
MFCC computation technique

Mel Frequency Cepstral Coefficient (MFCC) was employed as sound clip characteristics. Speech recognition systems frequently employ MFCCs. They have also been extensively employed in prior employment on the recognition of unexpected respiratory sound signals because they give an indication of the time domain short-term power spectrum of the sounds. Because multiple adventitious sounds might appear in the same tape at different periods and have varied durations, both frequency and time content are significant in distinguishing between them. As a result, MFCC is useful for recording a signal’s transform in frequency components during the time. Frequencies are allocated to the MEL scale that are nonlinear frequencies with equal distance in the human auditory system. Before further processing, MFCC generates a two-dimensional vector feature (frequency and time) that is compressed into an array of one-dimensional scale. The MFCC computation technique is depicted in Fig. 7.

## Elaboration studies

The studies [37, 38, 61] provided a survey of cutting-edge deep-learning-based respiratory nodule analysis and screening algorithms, with an emphasis on their presentation and medical applications. The study [61] compared the network performance, limitations, and potential trends of lung nodule investigation. The review [37] evaluated why molecular and cellular processes are of relevance. DLCNN has been used in different diagnostic procedures such as lung sound analysis, forced oscillation test, telemedicine, and breath analysis, with encouraging outcomes in small-scale investigations, according to [38].

In the same context, the papers [26, 48, 85, 91, 95, 136, 163] reviewed cancer diagnosis of the lung using medical picture analysis. Lung cancer is the foremost source of mortality globally, with “1.76 million related deaths recorded in 2018,” according to [26]. In addition, “Lung highest incidence rate of cancer death in both men and women, accounting for over a quarter of all cancer fatalities globally.” [48].

There are many published journal papers that review and proposed original methods to assess lung disease using deep learning CNN as an artificial intelligence technique. For highlighting the importance of these publications, this review briefly provides a table that lists the analyzed sample, the CNN algorithm type, tested data (sound or image samples), and their significant findings as seen in Table 4.

**Table 4:** List of the analyzed sample, the CNN algorithm type, tested data (sound or image samples), and their significant findings for the publications that have been surveyed

Study	Method	Splitting strategy	Performance			
			Specificity	Sensitivity	Accuracy	Score
Demir et al. [42, 43]	VGG16	Tenfold CV	–	–	63.09%	–
Serbes et al. [144]	SVM	Official 60/40	–	-	49.86%	–
Sen I, et al. [143]	GMM Classifier	–	90%	90%	85.00%	–
Saraiva et al. [141]	CNN	Random 70/30	–	–	74.3%	–
Yang et al. [172]	ResNet + SE + SA	Official 60/40	81.25%	17.84%	–	49.55%
Ma et al. [101]	bi-ResNet	Official 60/40 Random tenfold CV	69.20% 80.06%	31.12% 58.54%	52.79% 67.44%	50.16% 69.30%
Pham et al. [130]	CNN-MoE	Official 60/40 Random fivefold CV	68% 90%	26% 68%	–	47% 97%
Gairola et al. [55] official 60/40	CNN	Official 60/40 Interpatient 80/20	72.3% 83.3%	40.1% 53.7%	–	56.2% 68.5%
Liu et al. [91, 95]	CNN	Random 75/25	–	–	81.62%	–
Acharya and Basu [3]	CNN-RNN	interpatient 80/20	84.14%	48.63%	–	66.38%
Allahwardi & Altan et al. [14]	Deep Belief Networks (DBN)	–	93.65% 73.33%	93.34% 67.22%	95.84% 70.28%	
Kochetov et al. [80]	RNN	Interpatient fivefold CV	73%	58.4%	–	65.7%
Minami et al. [109]	CNN	Official 60/40	81%	28%	–	54%
Georgios Petmezas et al. [129]	CNN-LSTM with FL	Interpatient tenfold CV LOOCV	84.26% –	52.78% 60.29%	76.39% 74.57%	68.52% –
Chambres et al. [31]	HMM SVM	Official 60/40	56.69% 77.80%	42.32% 48.90%	49.50% 49.98%	39.37% 49.86%
Oweis et al. [122]	ANN	–	100%	97.8%	98.3%	–
Jakovljevi´c and Lonˇcar-Turukalo [73]	HMM	Official 60/40	–	–	-	39.56%
Bahoura [22]	GMM	–	92.8%	43.7%	80.00%	–
Emmanouilidou D et al. [46]	RBF SVM Classifier	–	86.55 (± 0.36)	86.82 (± 0.42)	86.70%	–
Ma et al. [98–100]	ResNet + NL	Official 60/40 Interpatient fivefold CV	63.20% 64.73%	41.32% 63.69%	–	64.21% 52.26%
Nangia et al. [21]	CNN	–	–	–	94.24%	93.6%
Pramono RX et al. [4]	SVM	–	83.86%	82.06%	87.18%	82.67%
Nguyen and Pernkopf [118]	ResNet	Official 60/40 Official 60/40	79.34% 82.46%	47.37% 37.24%	– 73.69%	58.29% 64.92%
Bardou D et al. [23]	CNN	–	–	–	95.56%	–
Aykanat M et al. [18]	ANN	–	86%	86%	76.00%	–
Chamberlain et al. [30]	–	–	0.56	–	86% Wheeze	–

The table shows a classification of some published articles and their achievements. The studies [1, 25, 74, 87] created a problem-based architecture that saves image data for identifying integration in a Chest Pediatric X-ray database. They designed a three-step pre-processing strategy to improve model generalization. An occlusion test is used to display model outputs and identify the observed relevant area in order to check the reliability of numerical findings. To test the universality of the proposed model, a different dataset is employed as additional validation. In real-world practice, the provided models can be used as computer-aided diagnosis tools. They thoroughly analyzed the datasets and prior studies based on them, concluding that the results could be misleading if certain precautions are not followed.

## Conclusions

This work provided a review of lung disease recognition with acoustic signal analysis with deep learning networks. Compared to related review studies on lung disease classification/detection using deep learning algorithms, only two review studies based on signal analysis for lung disease diagnosis have been conducted in 2011 and 2018. Deep Learning Convolutional Neural Networks (DLCNN) are being used to diagnose obstructive lung illnesses, which is a fascinating development. DLCNN algorithms function by identifying patterns in diagnostic test data that can be applied to forecast and identify obstructive phenotypes or clinical outcomes. DLCNN will require consensus examination, data analysis, and interpretation techniques as it matures as medical technology. To enable big clinical trials and, ultimately, minimize ordinary clinical use, such tools are required to compare, understand, and reproduce study findings from and among diverse research organizations. It is necessary to make recommendations on how DLCNN data might be used to generate diagnoses and influence clinical decision-making and therapeutic planning. This review looks at how deep learning can be used in medical diagnosis. A thorough assessment of several scientific publications in the field of deep neural network applications in medicine was conducted. More than 200 research publications were discovered, with 77 of them being presented in greater detail as a result of various selection techniques. Overall, the use of a DLCNN in the detection of obstructive lung disorders has yielded promising results. Large-scale investigations, on the other hand, are still needed to validate present findings and increase their acceptance by the medical community. We anticipate that physicians and researchers working with DLCNN, as well as industrial producers of this technology, will find this material beneficial.

## Data Availability

Not applicable because it's a review paper.
